# Benefits of robotic gait assistance with ATLAS 2030 in children with cerebral palsy

**DOI:** 10.3389/fped.2024.1398044

**Published:** 2024-07-29

**Authors:** Pilar Castro, María Martí, Bárbara Oliván-Blázquez, Nuria Boñar, Violeta García, Santiago Gascón-Santos, Alicia Panzano, Sara Vela, Sara Tajadura, Ana Peña, María Josefa Tris-Ara

**Affiliations:** ^1^ Asociación Tutelar Aragonesa de Discapacidad Intelectual (ATADES), Zaragoza, Spain; ^2^ Department of Psychology and Sociology, University of Zaragoza, Zaragoza, Spain; ^3^ Fundacion Bobath, Madrid, Spain; ^4^ Fundacion, Atenpace, Madrid, Spain; ^5^ Department of Paediatric Rehabilitation, Hospital Universitario Miguel Servet, Zaragoza, Spain

**Keywords:** Cerebral Palsy (CP), rehabilitation, Children, exoskeleton, therapy

## Abstract

**Objective:**

This study aims to assess the impact of integrating ATLAS 2030 into the conventional therapy regimen for children with Cerebral Palsy (CP) compared to conventional therapy alone regarding gross motor function, range of motion (ROM) and spasticity.

**Design:**

A non-randomized controlled trial conducted in outpatient rehabilitation settings and special education schools, following the recommendations by the Consolidated Standards of Reporting Trials (CONSORT) statement.

**Participants:**

Thirty children with CP divided into intervention and control groups.

**Intervention:**

The intervention group received three months of therapy (twice per week) with the ATLAS 2030 device in addition to their standard therapy, while the control group underwent standard therapy alone.

**Main outcome measure:**

Gross motor function assessed using the Gross Motor Function Measure of 88 items (GMFM-88).

**Secondary outcomes:**

Spasticity, measured by the Modified Ashworth Scale (MAS), and ROM of the lower limbs.

**Results:**

Statistically significant differences were observed between groups, in favour the intervention group, in both the GMFM-88 total score and dimension A, B and D. Similar findings were noted for spasticity and ROM, demonstrating significant improvements in the intervention group.

**Conclusion:**

ATLAS 2030 proves to be a safe and valuable tool for the rehabilitation of children with CP, showing improvements in motor function, spasticity and ROM.

## Introduction

Cerebral Palsy (CP) constitutes a collection of enduring movement and posture disorders, engendering activity limitations due to non-progressive lesions in the developing brain (1). The damage caused persists throughout life, giving rise to symptoms like spasticity (2), joint contractures (3), incoordination (4), and compromised selective motor control or weakness (5). All of this generates psychosocial and adaptation difficulties for the affected child and their families (6). Presently, CP afflicts approximately 2 cases per 1,000 births in Europe, standing as the principal cause of multifactorial motor disability in childhood (7). Spasticity frequently causes pain, gait disturbances, and mobility limitations. Over time, spasticity can lead to additional complications such as muscle spasms, joint contractures, difficulty moving in bed, trouble with transfers, poor seating posture, and impaired ability to stand and walk.

Depending on the topography, CP can be unilateral or bilateral. CP can be categorised also depending on the predominant motor disorder, it is classified as spastic, dyskinetic-dystonic, ataxic or mixed, being spastic CP the most frequent form (8). According to the severity of the impairment, a distinction is made between mild, moderate, severe or profound, or according to the functional level of mobility in levels I to V according to the Gross Motor Function Classification System (GMFCS) (9).

Existing evidence supports interventions grounded in functional training and physical activity, prioritizing specific exercises, and heightened active engagement as the most effective treatments for addressing motor function in CP (5). Physical activity in children with CP has been shown to enhance stability, balance, overall motor function, and aerobic capacity, consequently diminishing the risk of depression and fostering participation in daily activities. This includes diverse activities such as yoga, pilates, martial arts, cycling or robot-assisted gait training, depending on the possibilities of the user (10).

Despite the benefits conferred by such interventions, individuals with more severe disability, classified in levels IV and V of the GMFCS, face impaired walking ability. Even in levels I, II, or III, where walking ability is retained with or without technical aids, abnormal movement patterns may contribute to the development of secondary deformities. Hence, standing and gait training, along with other physiotherapy techniques and a multidisciplinary approach, emerge as crucial in the rehabilitation of this population (5).

Recent technological strides have ushered in robotic therapy within the neuro-rehabilitation realm, with exoskeletons standing out as a noteworthy engineering contribution to CP treatment. These devices exhibit moderate evidence but promise effectiveness with minimal adverse effects and ensured safety (11–15).

Gait exoskeletons, specifically, provide the opportunity to stand and walk safely, even to those patients with severe motor deficit, thanks to its powered structure attached to the user's body. These devices offer a symmetrical and cyclical kinematic gait pattern, thereby enhancing postural control and physical functions. Furthermore, they have the potential to delay or prevent associated organic, musculoskeletal, or psychosocial complications. In addition, exoskeleton training is anticipated to contribute not only to the improvement of physical variables but also to psychosocial aspects such as self-esteem, sociability, and integration, thereby enhancing quality of life (16, 17).

Whereas safety and feasibility of fixed and paediatric exoskeletons have been proved in children with CP (12) research on its effectiveness is still scarce, although recently published studies suggest improvements in gross motor function, walking speed or muscle activity. However, most of the published studies focused on fixed exoskeletons that walk on a treadmill, while research on overground devices is limited so far, with only a few published studies with small sample sizes and without control group (18–20).

Regarding dosage and frequency, most interventions are biweekly and duration ranges between 4 and 10 weeks observing good tolerance (18–20), although further research should be conducted in order to determine whether longer or more intensive interventions may provide larger improvements.

Therefore, given the lack of studies focusing on the efficacy of overground exoskeletons in the paediatric population with large sample sizes and control group comparisons (18), this study aims to evaluate the effectiveness of incorporating the ATLAS 2030 exoskeleton into conventional therapy for children with CP, comparing it to a group receiving only conventional therapy. The primary objective is to assess changes in gross motor function following a three-month intervention with the ATLAS 2030 in addition to the conventional therapy, with the expectation of improving gross motor function. Secondary objectives include measuring changes in spasticity and the range of motion (ROM) of the lower limbs, as improvements in these areas may lead to better movement capabilities and, consequently, better gross motor function.

## Methods

### Study design

**Figure 1:**
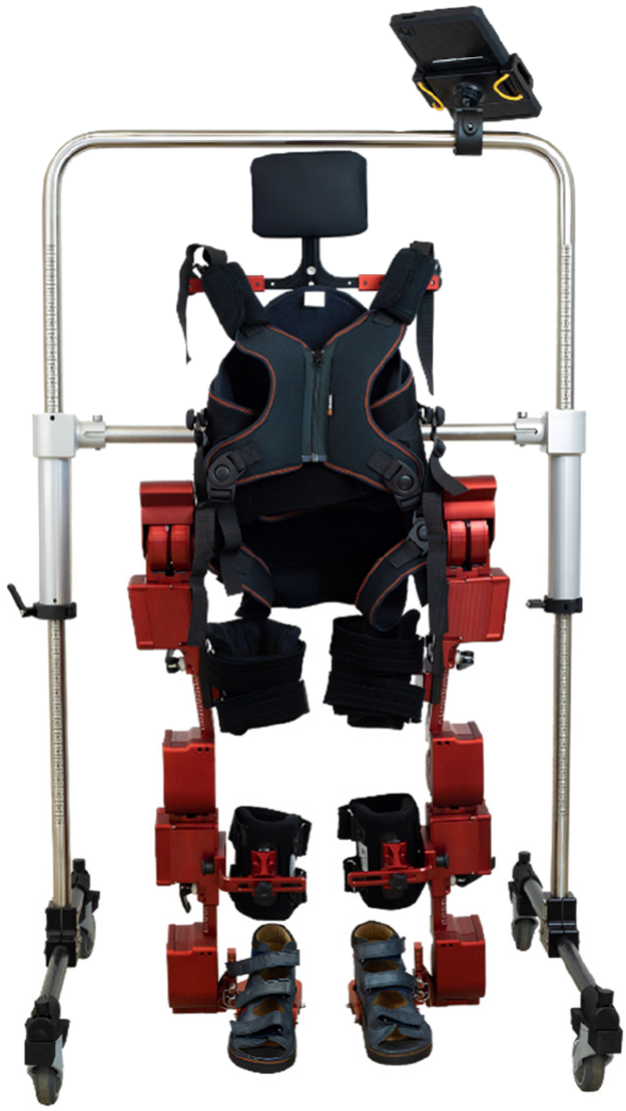
ATLAS 2030 exoskeleton.

A prospective non-randomized multi-centre study distributed in two parallel groups: a control group, in which participants received their usual conventional therapy for 12 weeks; and an intervention group in which participants followed also their usual conventional therapy and in addition received gait training sessions with the ATLAS 2030 exoskeleton (Figure 1) two times per week during the same period. Since the intervention was conducted in a small city, the access to a population of children with CP was limited. Therefore, the control group had to be recruited in a different city and in consequence, the study could not be conducted in a randomised fashion, as described below. Two equivalent groups in terms of pathology, demographics and standard treatment were used. This study was conducted in Spain from September 2022 to June 2023.

This study has been conducted in accordance with the standards of good clinical practice (ICH-GCP) and with the Declaration of Helsinki (21). It followed the recommendations established by the Consolidated Standards of Reporting Trials (CONSORT) statement (22, 23) for randomised controlled trials, and approval was obtained by the Ethics Committee of the Hospital to which the specialists who carried out the study belong, and of the National Agency for Medicines and Health Products of the country where the study was conducted (reference 1012/22/EC-R). The protocol of this study has previously been registered in clinicaltrials.gov (NCT05551364).

### Participants

Participants for the intervention group were recruited from ATADES, an education centre in Zaragoza, whereas the participants for the control group were recruited from two different rehabilitation centres in Madrid: Bobath Foundation and ATENPACE. All participants were recruited upon fulfilment of the main selection criteria, that were: a) confirmed diagnosis of CP; b) age 3–14 years (due to device size limitation); c) medical clearance for standing, gait training and weight bearing. The eligibility criteria were also the specifics of device use which are described in the study registered on clinicaltrials.gov with ID number NCT05551364 https://classic.clinicaltrials.gov/ct2/show/NCT05551364.

Participants were allocated to each group based on their respective rehabilitation centres. Those recruited from the centre with access to the ATLAS 2030 were assigned to the intervention group, while participants from centres without access to the device were assigned to the control group. In total, 30 children participated in the study, with 15 in each group.

Families in the control group were informed at the time of consent that they would have the opportunity to potentially benefit from exoskeleton training in the event of statistically significant benefits from the intervention, as indeed occurred.

#### Sample size

Based on the findings of Ko and Kim's study (24), with the GMFM-88 as the primary variable, sample size calculation was conducted using G*Power software (version 3.1.9.6). The study design dictated the following parameters: an effect size of 0.25, a correlation of 0.5 between repeated measurements, and assumptions of a 95% confidence interval and 80% power. Thus, a sample size of 24 individuals was determined. To account for potential loss to follow-up during the study, 25% of participants (*N* = 30) were added.

### Intervention

#### Intervention group

Over a 12-week period, the intervention group received their standard conventional therapy, maintaining the prescribed dosage and frequency throughout the study, and complemented by two additional weekly sessions utilizing the ATLAS 2030 exoskeleton. Led by a therapist certified in exoskeleton usage, each session lasted up to 60 min, primarily focusing on effective gait training for approximately 45 min.

The ATLAS 2030, a paediatric exoskeleton, is designed to enhance mobility for children with limited independent ambulation abilities. Classified as a class II-a medical device for external use, it holds certifications from both the European Community and the U.S. Food and Drug Administration (FDA).

This innovative exoskeleton facilitates both forward and backward walking, providing customized assistance tailored to individual needs. It operates in two modes: the automatic mode, which follows a predetermined walking pattern derived from healthy subjects' kinematics at a set speed, and the active-assisted mode, capable of detecting the user's movement intention and supplying necessary residual force. Moreover, the active-assisted mode offers the additional benefit of strengthening lower limb muscles.

**Figure 2:**
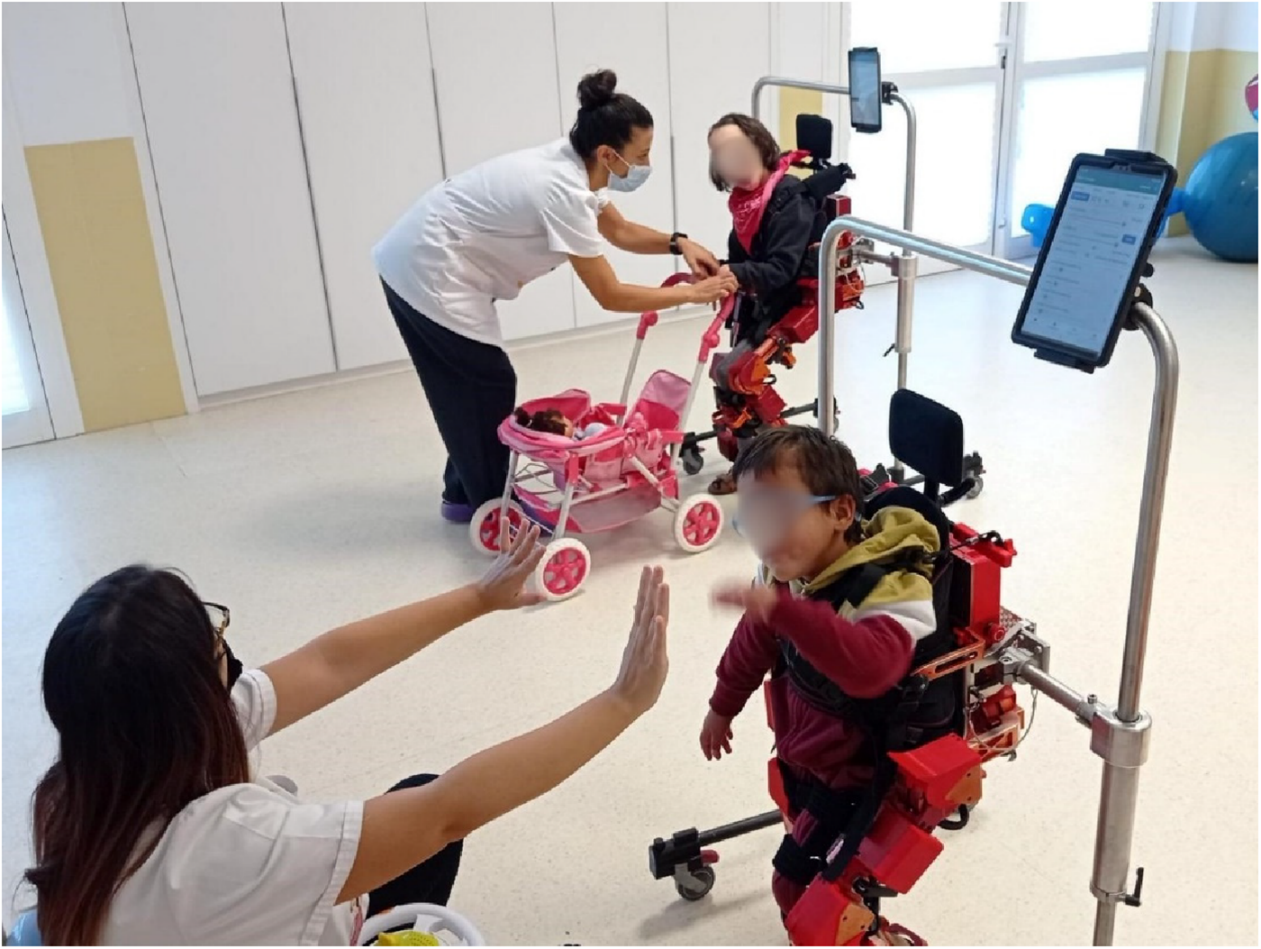
Children using ATLAS 2030 exoskeleton with rehabilitation specialists.

All modes of the ATLAS 2030 (automatic and active-assisted) were employed, with forward and backward walking tailored to each child's endurance level. Given its overground nature, the ATLAS 2030 allows therapists to have their hands free, enabling the incorporation of various games during sessions to address different rehabilitation objectives based on each participant's needs. Furthermore, the selection of usage modes was adjusted according to each participant's capabilities (Figure 2).

#### Control group

Throughout the 12-week study period, participants in the control group adhered to their regular conventional therapy sessions, maintaining the prescribed dosage and frequency. The conventional therapy regimen for both groups followed established clinical practice guidelines for CP (25, 26). This therapy emphasized mobility treatments using a functional task-based approach, including physical activity, postural management, stretching, strength training, standing, gait training, and goal-oriented training. Additionally, activities targeting cognition, sensory skills, and musculoskeletal development were incorporated, always ensuring that treatment objectives were pursued in a playful and motivating manner tailored to each child.

Both ATLAS 2030 and conventional therapy sessions were conducted by physical therapists specialized in neuro-rehabilitation with extensive experience in the field.

### Outcomes measures

#### Descriptive variables

Demographic data, including sex, age, and the level on the GMFCS (9, 27) were systematically gathered.

#### Measurements

Patients underwent evaluations at four distinct intervals: during the initial (baseline) session, after the first and second months, and upon completion of the intervention (at the third month). In addition, for the intervention group, an extra assessment of spasticity was conducted both before and after each device session to capture immediate changes.

To ensure consistency and reliability, all measurements were conducted by the same evaluator for each centre across all participants and measurements.

Assessment lasted between 1 and 2 h depending on the capacity of each participant to perform the tasks required to complete all tests.

#### Main outcome measures

The primary metric for assessment was gross motor function, as evaluated by the GMFM-88 (28). Tailored for children with CP aged between 5 months and 16 years, this observational scale comprises 88 items scored on a 4-point (0–3) scale, reflecting the child's proficiency in a specific task. These items are categorized into five dimensions, addressing distinct facets of motor skills: (A) Lying and Rolling (17 items), (B) Sitting (20 items), (C) Crawling and Kneeling (14 items), (D) Standing (13 items), and (E) Walking, Jumping, and Running (24 items). Each dimension is assessed on a scale of 0–100, with higher scores denoting superior gross motor function. This tool is linguistically translated and validated in Spanish (29), with a Cronbach's alpha of 0.936 obtained in this study.

#### Secondary outcome measures

To assess range of motion (ROM), three specific movements—hip extension, knee extension, and ankle dorsiflexion—were scrutinized, as these often exhibit limitations in children with CP. These limitations in ROM may result in mobility restrictions and, consequently, reduced independence in daily activities. A specialized physiotherapist utilized a manual goniometer, following the guidelines outlined by Norkin and White (30).

The Modified Ashworth Scale (MAS) (31) was employed to assess spasticity in the lower limbs. This scale, ranging from 0 to 4, indicates no spasticity at 0 and fixed joints at 4. Assessors evaluated spasticity in the hip flexors, knee extensors, and ankle plantarflexors, as these muscle groups are commonly affected in this population.

### Statistical methods

Clinical effectiveness was assessed using statistical analysis by protocol, since there was only one lost. First, the sample distribution was analysed, obtaining Shapiro–Wilk statistic values that were lower than 0.05 for all the variables, however, non-parametric statistics were used. A descriptive analysis of all the variables was carried out, using frequencies and percentages for categorical variables and median and interquartile range but also means and standard deviation for continuous variables. An initial comparison was made between both groups, examining key variables to establish the groups' baseline comparability. The differences between both groups related to clinical variables at baseline, three months and the improvements in each variable were calculated using Chi-squared tests for qualitative variables, such as sex and GMFCS, and Mann-Whitney U for the rest of the variables.

Finally, in order to analyse the variables associated with effectiveness, a linear regression was performed considering the difference in the score at 3 months and at baseline for the main variable, it means, the improvements in GMFM-88 and their five dimensions. The independent variables were added into the regression model, and a final model was obtained. Linear regression was used since the residuals of the model had a finite mean, constant variance, and normal distribution. However, bootstrapping analysis with 2,000 samples was also conducted. The dependent variable was the improvement in the GMFM-88 (total score and dimensions). The independent variables were the treatment group (intervention group or control group), age and GMFCS.

## Results

### Participant flow and compliance

**Figure 3:**
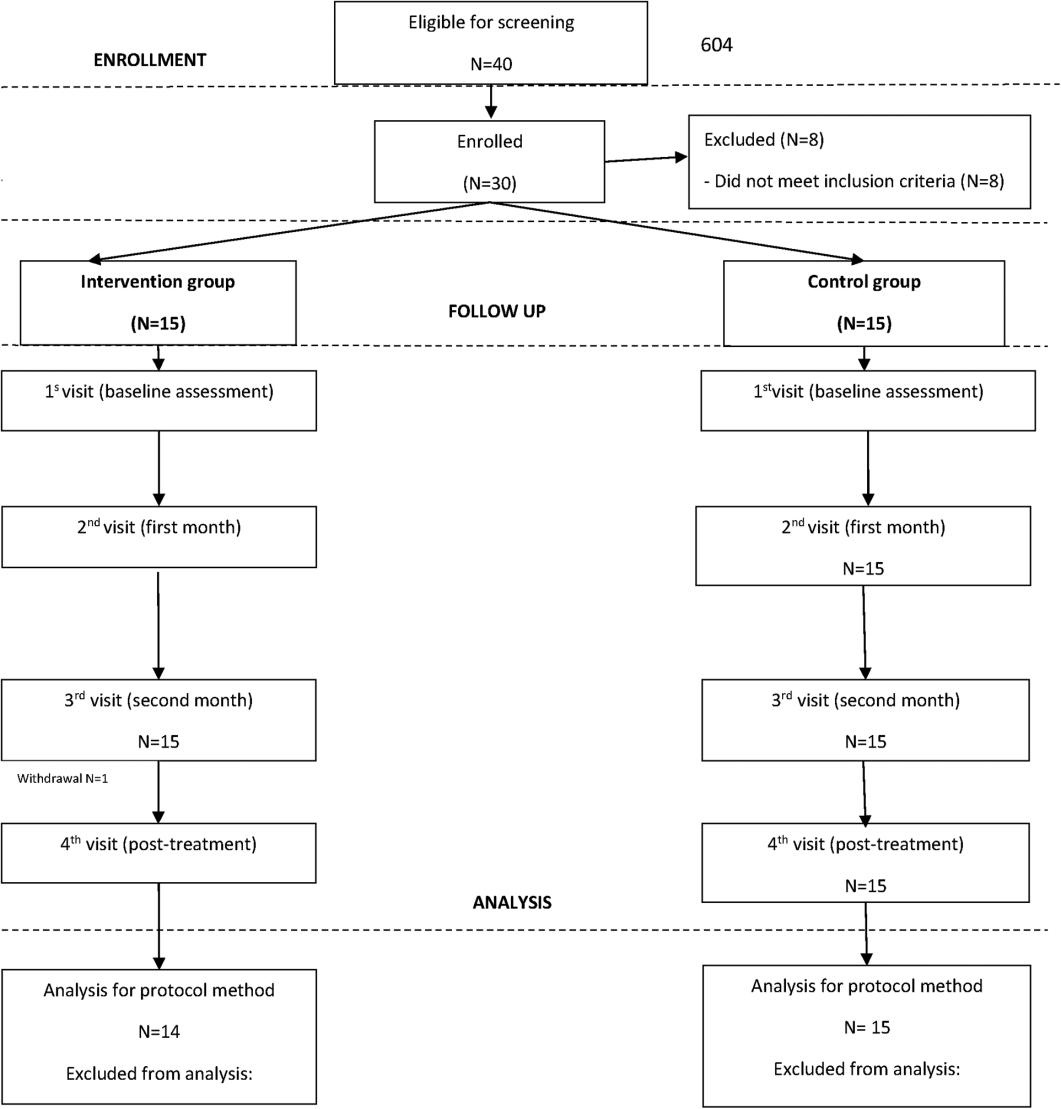
Flow chart of participants during the trial.

Figure 3 illustrates the participant flow throughout the trial. Post-treatment assessments were completed by 14 (93.33%) children in the intervention group and all 15 (100%) children in the control group. The sole dropout in the intervention group resulted from a non-CP-related illness. Due to the minimal dropout rate, further analysis of predictors for dropout was deemed unnecessary.

### Group baseline characteristics and groups comparison

Table 1 delineates the baseline characteristics of the entire sample and provides a comparison of both groups. The cohort consisted of 30 children, with 11 girls and 19 boys, and a median age of 6.12 (IQR 3.54). Half of the children were classified at level V on the GMFCS, showing spasticity assessed by the MAS with values between 1 and 2 in hips, knees and ankles. In terms of the GMFM-88 score, the participating children had higher scores in the dimensions Lying and Balancing, and Sitting, with a subsequent decrease in scores in the Crawling and Kneeling, Standing, and Walking, Jumping and Running dimensions.

**Table 1:** Description of the baseline characteristics of the total sample and contrast of the intervention and control group.

	Total sample *N* = 30	Intervention group *N* = 15	Control group *N* = 15	*p*-value (CI)
Gender (%, *n*)	36.6 F (11), 63.3 M (19)	40% F (6), 60% M (9)	33.33% F (5), 66.66 M (10)	0.705
Age (mean SD; median IQR)	6.49 (2.41)/6.12 (3.54)	5.67 (1.95)/5.57 (2.87)	7.30 (2.60)/7.23 (4.81)	0.106 (−3.40; 0.23)
GMFCS (*n* %)				
Level 1	0 (0)	0 (0)	0 (0)	0.751
Level 2	4 (13.3)	2 (13.3)	2 (13.3)	
Level 3	4 (13.3)	1 (6.7)	3 (20.0)	
Level 4	7 (23.3)	4 (26.7)	3 (20.0)	
Level 5	15 (50.0)	8 (53.3)	7 (46.7)	
GMFM-88 (mean SD; median IQR)				
A: Lying and Rolling	71.37 (27.48)/79.41 (39.7)	69.28 (32.11)/82.35 (37.25)	73.46 (22.88)/76.47 (41.17)	0.967 (−17.64;19.60)
B: Sitting	53.22 (34.44)/65 (72.51)	50.55 (33.74)/61.66 (68.33)	55.88 (36.10)/70.00 (71.67)	0.539 (−26.66; 21.66)
C: Crawling and Kneeling	38.09 (31.83)/38.09 (62.5)	38.25 (31.87)/35.71 (59.52)	37.93 (32.91)/42.85 (64.28)	1.00 (−26.19;28.57)
D: Standing,	20.17 (24.75)/8.97 (26.28)	16.23 (22.69)/7.69 (23.07)	24.10 (26.88)/15.38 (46.16)	0.389 (−17.94; 5.12)
E: Walking Jumping and Running	15.0 (21.41)/4.86 (18.05)	10.46 (20.47)/0.00 (16.66)	19.53 (22.06)/12.50 (40.27)	0.089 (−13.88;0.01)
Total score	39.58 (25.31)/39.11 (44.53)	36.95 (24.97)/36.67 (42.47)	42.21 (26.23)/46.57 (46.70)	0.595 (−24.53;14.72)
ROM (mean SD; median IQR)				
Hip extension	8.19 (9.71)/8.25 (12.25)	11.75 (10.03)/14.25 (13.12)	4.64 (8.25)/5 (8.12)	0.044 (0.5;14)*
Knee extension	−1.78 (5.07)/0 (7.25)	−1.75 (6.05)/−1 (11.5)	−1.82 (4.10)/0 (0.75)	0.874 (−5; 4.5)
Ankle dorsiflexion	−2.15 (13.64)/0 (15.5)	−1.33 (14.72)/−5 (22.5)	−3.03 (12.86)/0 (4.37)	0.813 (−9;11)
MAS (mean SD; median IQR)				
Hip flexion	1.12 (0.89)/1.25 (1.75)	1.28 (0.84)/1.25 (1.5)	0.96 (0.93)/1.00 (1.5)	0.354 (−0.50; 1.00)
Hip extension	0.94 (0.79)/1.00 (1.5)	1.14 (0.94)/1.25 (1.81)	0.76 (0.59)/1.00 (1)	0.217 (0.00; 1.00)
Knee flexion	1.05 (0.91)/1.00 (1.62)	1.42 (0.79)/1.50 (1)	0.70 (0.90)/0.00 (1.5)	0.018 (0.00; 1.50)*
Knee extension	0.78 (0.85)/1.00 (1.12)	1.05 (1.00)/1.00 (1.56)	0.53 (0.61)/0.00 (1)	0.186 (0.00;1.00)
Ankle flexion	1.20 (1.08)/1.50 (1.75)	1.30 (1.37)/1.18 (2.25)	1.11 (1.02)/1.50 (1.5)	0.621 (−0.75;1.25)
Ankle extension	1.20 (1.01)/1.00 (1.75)	1.41 (1.37)/1.15 (2.62)	1.01 (0.85)/1.00 (1.5)	0.400 (−0.50;1.50)

GMFCS, gross motor function classification system; GMFM-88, gross motor function measure of 88 items; ROM, range of motion; MAS, modified ashworth scale; CI, confidence interval.

Statistics used: Except of the gender and GMFCS which are show in frequency and percentage, all the rest of the variables are shown in mean SD/median IQR. Chi-square and Mann-Whitney U for comparison. Confidence interval: Hodges-Lehman median difference for independent samples.

*p-value < 0.05.

Statistically significant differences between groups are observed in the measurement of knee flexion spasticity and the ROM related to hip extension (both right and left), being higher the ROM in hip extension in the intervention group but also the knee flexion spasticity.

### Main and secondary outcome measures

**Figure 4:**
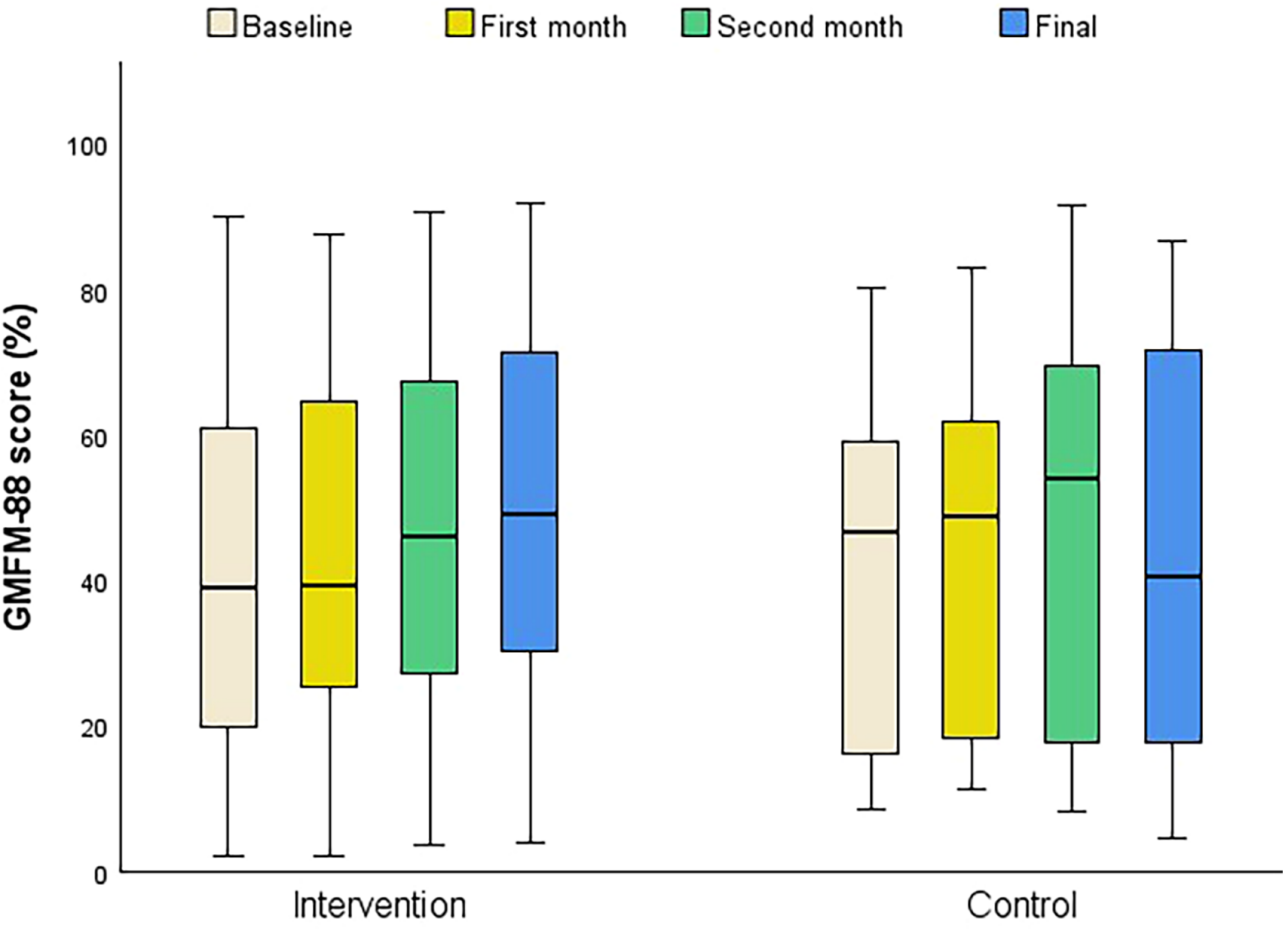
Progression of GMFM-88 total score over months. Results are included for all participants who completed all study assessments (intervention *n* = 14, control *n* = 15).

Table 2 presents comparative analysis, including means and standard deviations, as well as medians and interquartile ranges (IQRs). Significance measures, such as *p*-values and confidence intervals, are provided for basal, post-treatment, and improvement values in each variable. Children undergoing conventional therapy and ATLAS 2030 exhibited noteworthy improvements compared to the control group in the GMFM-88 total score and the dimensions of Lying and Rolling, Sitting, and Standing. The intervention group demonstrated significant enhancements in all ROM measurements (hip extension, knee extension, and ankle dorsiflexion for both legs) and in most of spasticity evaluations (hip flexion and extension, knee flexion and extension, and ankle dorsiflexion). Figure 4 shows the progression of each group across each evaluation.

**Table 2:** Outcome variables at basal, post-treatment and improvements between intervention and control groups.

	Intervention group *N* = 14	Control group *N* = 15	*p*-value (CI)
GMFM-88 (mean SD; median IQR)			
A: Lying and rolling			
Baseline	69.28 (32.11)/82.35 (37.25)	73.46 (22.88)/76.47 (41.17)	0.967 (−17.64;19.60)
Post-treatment	83.05 (31.29)/95.09 (14.71)	72.02 (23.38)/74.50 (25.49)	0.026 (1.96;29.41)*
Improvement	10.78 (11.40)/7.84 (15.70)	−1.43 (10.77)/0.00 (13.72)	0.016 (1.96;19.60)*
B: Sitting			
Baseline	50.55 (33.74)/61.66 (68.33)	55.88 (36.10)/70.00 (71.67)	0.539 (−26.66; 21.66)
Post-treatment	70.11 (31.86)/77.50 (57.92)	60.88 (35.48)/60.00 (73.33)	0.505 (−13.33;40.00)
Improvement	16.54 (12.06)/12.50 (15.42)	5.00 (16.99)/3.33 (11.66)	0.008 (3.33;15.00)**
C: Crawling and kneeling			
Baseline	38.25 (31.87)/35.71 (59.52)	37.93 (32.91)/42.85 (64.28)	1.00 (−26.19;28.57)
Post-treatment	50.17 (53.57)/34.83 (62.5)	42.53 (40.13)/57.14 (80.95)	0.652 (−16.66;45.23)
Improvement	9.52 (9.05)/7.14 (14.88)	42.53 (40.13)/57.14 (16.66)	0.102 (−2.38;14.28)
D: Standing			
Baseline	16.23 (22.69)/7.69 (23.07)	24.10 (26.88)/15.38 (46.16)	0.389 (−17.94; 5.12)
Post-treatment	27.47 (30.19)/17.94 (41.67)	26.32 (31.09)/15.38 (64.10)	0.652 (−17.94;17.94)
Improvement	10.07 (17.25)/6.41 (10.89)	2.22 (8.04)/−2.56 (12.81)	0.057 (0.00;10.25)*
E: Walking jumping and running			
Baseline	10.46 (20.47)/0.00 (16.66)	19.53 (22.06)/12.50 (40.27)	0.089 (−13.88;0.01)
Post-treatment	15.77 (22.62)/1.38 (28.81)	23.98 (27.52)/13.88 (61.12)	0.310 (−19.44; 5.55)
Improvement	4.56 (8.12)/0.00 (10.06)	4.44 (8.13)/0.00 (8.3)	0.914 (−4.16;4.16)
Total score			
Baseline	36.95 (24.97)/36.67 (42.47)	42.21 (26.23)/46.57 (46.70)	0.595 (−24.53;14.72)
Post-treatment	49.31 (26.63)/49.23 (42.14)	45.15 (29.28)/40.57 (61.63)	0.715 (−16.92;29.65)
Improvement	10.29 (6.89)/9.55 (7.56)	2.93 (7.65)/2.47 (7.78)	0.012 (1.93;11.43)**
ROM (mean SD; median IQR)			
Hip extension			
Baseline	11.75 (10.03)/14.25 (13.12)	4.64 (8.25)/5 (8.12)	0.044 (0.5;14)*
Post-treatment	19.67 (7.21)/20.5 (9.12)	5 (6.57)/5 (8.12)	<0.001 (10;20)**
Improvement	6.11 (7.70)/3.5 (12.75)	0.35 (2.16)/0 (0)	0.048 (0;12.5)*
Knee extension			
Baseline	−1.75 (6.05)/−1 (11.5)	−1.82 (4.10)/0 (0.75)	0.874 (−5; 4.5)
Post-treatment	1.85 (6.97)/3.75 (12.87)	−2 (4.51)/0 (0.75)	0.227 (−3;8.5)
Improvement	3.84 (4.72)/5 (4.25)	−0.17 (0.66)/0 (0)	0.005 (2.5;5)*
Ankle dorsiflexion			
Baseline	−1.33 (14.72)/−5 (22.5)	−3.03 (12.86)/0 (4.37)	0.813 (−9;11)
Post-treatment	7.35 (5.99)/7.5 (8.87)	−5.35 (11.47)/0 (10.62)	<0.001 (5;15.5)**
Improvement	8.42 (15.07)/4 (22.5)	−2.32 (7.36)/0 (0)	0.027 (1;18.5)*
MAS (mean SD; median IQR)			
Hip flexion			
Baseline	1.28 (0.84)/1.25 (1.5)	0.96 (0.93)/1.00 (1.5)	0.354 (−0.50; 1)
Post-treatment	0.32 (0.51)/0 (1)	1.10 (1.10)/1 (2)	0.072 (−1.5;0)
Improvement	−0.85 (0.63)/−0.87 (1.12)	0.13 (0.44)/0 (0)	<0.001(−1.5;0-.5)**
Hip extension			
Baseline	1.14 (0.94)/1.25 (1.81)	0.76 (0.59)/1.00 (1)	0.217 (0; 1)
Post-treatment	0.28 (0.47)/0 (0.75)	1.03 (0.91)/1 (1.5)	0.029 (−1.5;0)*
Improvement	−0.81 (0.73)/−0.75 (1.68)	0.26 (0.62)/0 (0.5)	0.001 (−1.75;−0.5)**
Knee flexion			
Baseline	1.42 (0.79)/1.50 (1)	0.70 (0.90)/0.00 (1.5)	0.018 (0; 1.5)*
Post-treatment	0.21 (0.43)/0 (0.25)	0.86 (1.10)/0 (1.5)	0.170 (−1;0)
Improvement	−1.18 (0.71)/−1.25 (0.87)	0.16 (0.55)/0 (0)	<0.001 (−1.75;−1)**
Knee extension			
Baseline	1.05 (1.00)/1.00 (1.56)	0.53 (0.61)/0.00 (1)	0.186 (0;1)
Post-treatment	0.50 (0.54)/0.5 (1)	0.83 (0.93)/1 (1.5)	0.440 (−1;0)
Improvement	−0.50 (0.73)/−0.25 (0.17)	0.30 (0.59)/0 (0.5)	0.012 (−1;0)**
Ankle dorsiflexion			
Baseline	1.30 (1.37)/1.18 (2.25)	1.11 (1.02)/1.50 (1.5)	0.621 (−0.75;1.25)
Post-treatment	0.15 (0.37)/0 (0)	1.15 (1.17)/1 (2)	0.022 (−1.5;0.)*
Improvement	−1.16 (1.17)/−1.12 (2)	0.03 (0.51)/0 (0)	0.010 (−2;0)**
Ankle plantar flexion			
Baseline	1.41 (1.37)/1.15 (2.62)	1.01 (0.85)/1.00 (1.5)	0.400 (−0.5;1.5)
Post-treatment	1.51 (0.34)/1.50 (0.62)	1.13 (1.04)/1 (2)	0.170 (−0.25;1.25)
Improvement	0.08 (0.25)/1.26 (2.18)	0.11 (0.64)/0 (0)	0.943 (−1;1)

GMFCS, gross motor function classification system; GMFM-88, gross motor function measure of 88 items; CPQLQ, cerebral palsy quality of life questionnaire; Weefim scale: Functional Independence Measure for Children; ROM, range of motion; MAS, modified Ashworth scale; CI, confidence interval. Statistics used: mean (SD)/median (IQR). Mann-Whitney U for comparison. Confidence interval: Hodges-Lehman median difference for independent samples.

*p-value < 0.05; **p-value ≤ 0.01.

The differences between means and medians, that are shown in Table 2, would imply that there are distinct profiles of children that have experienced varying degrees of improvement in the study. In the conducted multivariate analysis developed to analyze this profile, (Table 3), significant models were derived concerning the main variable, the GMFM-88 total score (*p* < 0.001), and subcategories including Lying and Rolling (*p* = 0.005), Sitting (*p* = 0.001), Crawling and Kneeling (*p* = 0.008), and Walking, Running, and Jumping (*p* = 0.039). The model associated with the Standing category yielded a *p*-value of 0.080. R-squares in these models ranged from 0.527 to 0.233. As detailed in Table 3, the analysis revealed that being part of the intervention group and older age were identified as significant factors associated with improvements in the total score, as well as the domains of Lying and Rolling, Sitting, and Crawling and Kneeling. Additionally, the GMFCS variable was associated with an improvement in the dimensions of Sitting and Walking, Running, and Jumping.

**Table 3:** Linear regression models with regard to improvements in the GMFM-88 (total score and the five dimensions).

GMFM-88 total score	Coefficient	*p*-value	Confidence interval 95%		Colinearity	
			Inferior	Superior	Tolerance	VIF
Constant	−12.760	0.040*	−24.878	−0.643		
GMFCS	0.079	0.937	−1.949	2.106	0.958	1.044
Age	2.104	<0.001**	1.137	3.071	0.845	1.183
Intervention Group	10.892	<0.001**	6.315	15.470	0.878	1.138
R^2^	0.571					
R^2^_adj_	0.519					
A: Lying and Rolling	Coefficient	*p*-value	Confidence interval 95%		Colinearity	
			Inferior	Superior	Tolerance	VIF
Constant	−12.760	0.022*	−48.625	−4.083		
GMFCS	0.079	0.154	−1.069	6.382	0.958	1.044
Age	2.104	0.032*	0.178	3.732	0.845	1.183
Intervention group	10.892	0.001**	6.718	23.545	0.878	1.138
R2	0.396					
R2adj	0.324					
B: Sitting	Coefficient	*p*-value	Confidence interval 95%		Colinearity	
			Inferior	Superior	Tolerance	VIF
Constant	−41.020	0.004**	−67.482	−14.558		
GMFCS	5.174	0.024*	0.747	9.600	0.958	1.044
Age	3.465	0.002*	1.353	5.577	0.845	1.183
Intervention group	16.637	0.002*	6.640	26.634	0.878	1.138
R2	0.457					
R2adj	0.392					
C: Crawling and kneeling	Coefficient	*p*-value	Confidence interval 95%		Colinearity	
			Inferior	Superior	Tolerance	VIF
Constant	−7.528	0.457	−28.055	13.000		
GMFCS	−1.585	0.351	−5.019	1.849	0.958	1.044
Age	2.527	0.004**	0.889	4.165	0.845	1.183
Intervention group	9.398	0.020**	1.643	17.153	0.878	1.138
R2	0.369					
R2adj	0.293					
D: Standing	Coefficient	*p*-value	Confidence interval 95%		Colinearity	
			Inferior	Superior	Tolerance	VIF
Constant	4.042	0.763	−23.327	31.412		
GMFCS	−3.122	0.172	−7.700	1.456	0.958	1.044
Age	1.460	0.181	−0.725	3.644	0.845	1.183
Intervention group	10.752	0.042*	0.412	21.092	0.878	1.138
R2	0.233					
R2adj	0.141					
E: Walking, running, and jumping	Coefficient	*p*-value	Confidence interval 95%		Colinearity	
			Inferior	Superior	Tolerance	VIF
Constant	7.059	0.357	−8.439	22.558		
GMFCS	−2.665	0.044*	−5.258	−0.073	0.958	1.044
Age	1.101	0.079	−0.136	2.338	0.845	1.183
Intervention group	2.352	0.416	−3.503	8.207	0.878	1.138
R2	0.280					
R2adj	0.194					

*p-value < 0.05; **p-value ≤ 0.01.

## Discussion

The results of this study indicate significant improvements in physical function in children with CP following the integration of the ATLAS 2030 training program with conventional therapy, as evidenced by the increase in the total Gross Motor Function Measure-88 (GMFM-88) score, the primary outcome of this research. Despite participants maintaining consistent dosage and type of conventional therapy throughout the study, variability in dosage and techniques cannot be entirely excluded. Nevertheless, participants who received the ATLAS 2030 intervention demonstrated greater improvements compared to those who continued with standard treatment alone. This enhanced improvement may be attributed to the increased stimulation provided by the ATLAS 2030, particularly for children with higher levels of impairment. The device supports proper body alignment, including trunk and head positioning, and ensures safety, enabling therapists to work directly in front of the child while allowing the children to use their hands for various activities. Consequently, the device facilitates simultaneous targeting of multiple treatment goals. Future research is required to evaluate the impact of exclusively using ATLAS 2030 or its combination with specific dosages and treatment regimens.

These positive effects extend to specific dimensions of the GMFM-88, including Lying and Rolling, Sitting, and Standing, and are shown even in children with severe disability. Studies focusing on participants at GMFCS levels II and III demonstrated advancements in more intricate motor dimensions (D and E), possibly due to the participants' ability to walk independently. Notably, no studies were found reporting results for participants categorized as levels IV and V according to the GMFCS. This absence may be attributed to the current lack of above-ground paediatric exoskeletons suitable for individuals severely affected at a motor level within this population (18).

As stated in the introduction, physical activity has shown to be beneficial for children with CP (10). Nevertheless, children classified at higher levels (IV-V) on the GMFCS face substantial limitations in their ability to engage in conventional physical activities. Exercising for these individuals entails considerable demands and additional efforts. In this regard, ATLAS 2030 emerges as a valuable rehabilitation tool, providing this population with the opportunity to engage in physical exercise concurrently with walking and playing. Moreover, this physical activity can be seamlessly incorporated into the environment, thereby aligning with recommendations in the scientific literature to enhance motor performance (5, 26, 32).

It is essential to underscore that an increase in the GMFM-88 score not only indicates improvements in motor functions but also suggests potential positive impacts on overall functionality and quality of life. This progress could translate into heightened engagement in daily activities, a reduction in functional limitations, and can contribute to bolstering self-esteem and psychological well-being for both patients and their families. Consequently, advancements in these areas represent not only strides in motor skills but also wield substantial influence on the overall quality of life, daily participation, and emotional well-being of children with CP (10, 33). This influence on and quality of life should be measured and analysed in further studies conducted with overground exoskeletons.

The regression analysis highlights the significant influence of age and the intervention group on various components of the GMFM-88 total score. Positive coefficients associated with the intervention group underscore the potential added value of integrating ATLAS 2030 into conventional therapy for enhancing motor function in children with CP across different activities. Further research is needed to explore variables that may impact participants' score progressions. Regarding the influence of age, no relationship between age and improvements in gross motor function after a rehabilitation program has been established in the scientific literature, although it might be related to a better cognitive level to understand and follow the commands required in the GMFM-88, influencing the results.

Improvements in gross motor function could be partially attributed to advancements achieved in secondary variables of ROM and spasticity (34). Significant enhancements were noted in all assessed ROM, particularly affected by joint restrictions in individuals with CP, including hip extension, knee extension, and ankle dorsiflexion. These findings align with other published case series involving children with CP and Spinal Muscular Atrophy, offering a more substantial sample size and comparisons with a control group (12, 35).

In terms of spasticity, the intervention group experienced statistically significant reductions, except for the ankle extension, which exhibited no noticeable changes between groups. The observed enhancements in both spasticity and ROM likely contribute to improved motor function in children, facilitating smoother and more coordinated movements. These results are consistent with other case series involving overground exoskeleton studies with children with CP. In contrast, studies on treadmill exoskeletons by Amman-Reiffer et al. (36), and Digiacomo et al. (37) found no significant differences in spasticity when comparing conventional physiotherapy with the Lokomat exoskeleton. Similarly, Peri et al. (38) reported no variations in spasticity among different treatment approaches, including conventional physiotherapy and using the Lokomat. However, the studies were not homogeneous in terms of dosage or the level of disability of the subjects, so further research should be conducted to compare the effects of overground exoskeletons vs. fixed exoskeletons in spasticity, among other variables.

### Study limitations

While this study offers valuable insights into the effectiveness of the combined intervention, it's essential to acknowledge some limitations. Difficulty in assembling a large sample at a single centre precluded randomization of participant assignment to the intervention or control group. Consequently, baseline differences in GMFM-88 scores were observed between the groups, potentially influenced by the non-random assignment process. Additionally, assessments throughout the study were not blinded, introducing the possibility of observer bias. The aim of this study was to assess the intervention of adding ATLAS 2030 to the current rehabilitation treatment that the participants were already receiving what it could drive to a limitation related to treatment homogeneity.

## Conclusion

The combination of ATLAS 2030 and conventional therapy has shown to be more beneficial than conventional therapy alone in improving gross motor function, spasticity, and ROM in children with CP. These results underscore the potential value of ATLAS 2030 as a valuable tool for the rehabilitation of this population.

These findings open avenues for future research, urging a deeper investigation into the long-term impact, functional outcomes, and improvements in quality of life associated with the ATLAS 2030 intervention.

## Data Availability

The raw data supporting the conclusions of this article will be made available by the authors, without undue reservation.

## References

[1] Aisen ML Kerkovich D Mast J Mulroy S Wren TA Kay RM Cerebral palsy: clinical care and neurological rehabilitation. Lancet Neurology. (2011) 10(9):844–52. 10.1016/S1474-4422(11)70176-4 21849165

[2] Wahyuni LK. Multisystem compensations and consequences in spastic quadriplegic cerebral palsy children. Front Neurol. (2023) 13:1076316. 10.3389/fneur.2022.1076316 36698899 PMC9868261

[3] Fitoussi F Dudek PL. The upper limb in children with cerebral palsy. Evaluation and treatment. Orthop Traumatol Surg Res. (2024) 110(1 Suppl):103763. https://doi.org.cuarzo.unizar.es:9443/ 10.1016/j.otsr.2023.103763 37992866

[4] Abdelhaleem N El Wahab MSA Elshennawy S. Effect of virtual reality on motor coordination in children with cerebral palsy: a systematic review and meta-analysis of randomized controlled trials. Egypt J Med Hum Genet. (2022) 23(1):71. 10.1186/s43042-022-00258-0

[5] Novak I Morgan C Fahey M Finch-Edmondson M Galea C Hines A State of the evidence traffic lights 2019: systematic review of interventions for preventing and treating children with cerebral palsy. Curr Neurol Neurosci. (2020) 20(3):1–21. 10.1007/s11910-020-1022-z 32086598 PMC7035308

[6] Parkes J White-Koning M Dickinson HO Thyen U Arnaud C Beckung E Psychological problems in children with cerebral palsy: a cross-sectional European study. J Child Psychol Psychiatry. (2008) 49(4):405–13. 10.1111/j.1469-7610.2007.01845.x 18081767

[7] Arnaud C Ehlinger V Perraud A Kinsner-Ovaskainen A Klapouszczak D Himmelmann K Public health indicators for cerebral palsy: a European collaborative study of the surveillance of cerebral palsy in Europe network. Paediatr Perinat Epidemiol. (2023) 37(5):404–12. 10.1111/ppe.12950 36722642

[8] Reid SM Carlin JB Reddihough DS. Classification of topographical pattern of spasticity in cerebral palsy: a registry perspective. Res Dev Disabil. (2011) 32(6):2909–15. https://doi: 10.1016/j.ridd.2011.05.012 21624819

[9] Palisano R Rosenbaum P Bartlett D Livingston M Walter S Russell D. Gross Motor Function Classification System: Expanded and Revised. GMFCS-E&R. Vol. 7. Hamilton: CanChild Centre for Childhood Disability Research, McMaster University (2007). p. 1–6.

[10] Selph SS Skelly AC Wasson N Dettori JR Brodt ED Ensrud E Physical activity and the health of wheelchair users: a systematic review in multiple sclerosis, cerebral palsy, and spinal cord injury. Arch Phys Med Rehabil. (2021) 102(12):2464–81. 10.1016/j.apmr.2021.10.002 34653376

[11] Bunge LR Davidson AJ Helmore BR Mavrandonis AD Page TD Schuster-Bayly TR Effectiveness of powered exoskeleton use on gait in individuals with cerebral palsy: a systematic review. PLoS One. (2021) 16(5):e0252193. 10.1371/journal.pone.0252193 34038471 PMC8153467

[12] Cumplido-Trasmonte C Ramos-Rojas J Delgado-Castillejo E Garcés-Castellote E Puyuelo-Quintana G Destarac-Eguizabal MA Effects of ATLAS 2030 gait exoskeleton on strength and range of motion in children with spinal muscular atrophy II: a case series. J NeuroEngineering Rehabil. (2022) 19:75. 10.1186/s12984-022-01055-x PMC9297544 35854321

[13] Llamas-Ramos R Sánchez-González JL Llamas-Ramos I. Robotic systems for the physiotherapy treatment of children with cerebral palsy: a systematic review. Int J Environ Res Public Health. (2022) 19(9):5116. 10.3390/ijerph19095116 35564511 PMC9100658

[14] Moll F Kessel A Bonetto A Stresow J Herten M Dudda M Use of robot-assisted gait training in pediatric patients with cerebral palsy in an inpatient setting-A randomized controlled trial. Sensors (Basel, Switzerland). (2022) 22(24):9946. 10.3390/s22249946 36560316 PMC9783925

[15] Wang Y Zhang P Li C. Systematic review and network meta-analysis of robot-assisted gait training on lower limb function in patients with cerebral palsy. Neurol Sci. (2023) 44(11):3863–75. 10.1007/s10072-023-06964-w 37495708 PMC10570202

[16] Hunt M Everaert L Brown M Muraru L Hatzidimitriadou E Desloovere K. Effectiveness of robotic exoskeletons for improving gait in children with cerebral palsy: a systematic review. Gait Posture. (2022) 98:343–54. 10.1016/j.gaitpost.2022.09.082 36306544

[17] Maher CA Toohey M Ferguson M. Physical activity predicts quality of life and happiness in children and adolescents with cerebral palsy. Disabil Rehabil. (2016) 38(9):865–9. 10.3109/09638288.2015.1066450 26218617

[18] Cumplido C Delgado E Ramos J Puyuelo G Garcés E Destarac MA Gait-assisted exoskeletons for children with cerebral palsy or spinal muscular atrophy: a systematic review. NeuroRehabilitation. (2021) 49(3):333–48. 10.3233/NRE-210135 34219676

[19] Yoo M Ahn JH Park ES. The effects of over-ground robot-assisted gait training for children with ataxic cerebral palsy: a case report. Sensors (Basel, Switzerland). (2021) 21(23):7875. 10.3390/s21237875 34883877 PMC8659941

[20] Kim SK Park D Yoo B Shim D Choi JO Choi TY Overground robot-assisted gait training for pediatric cerebral palsy. Sensors (Basel, Switzerland). (2021) 21(6):2087. 10.3390/s21062087 33809758 PMC8002375

[21] World Medical Association. World medical association declaration of Helsinki: ethical principles for medical research involving human subjects. JAMA. (2013) 310:2191–4. 10.1001/jama.2013.281053 24141714

[22] Calvert M Blazeby J Altman DG Revicki DA Moher D Brundage MD. Reporting of patient-reported outcomes in randomized trials: the CONSORT PRO extension. JAMA. (2013) 309:814–22. 10.1001/jama.2013.879 23443445

[23] Moher D Hopewell S Schulz KF Montori V Gøtzsche PC Devereaux PJ CONSORT 2010 Explanation and elaboration: updated guidelines for reporting parallel group randomised trials. Br Med J. (2010) 340:c869. https://doi.org.cuarzo.unizar.es:9443/10.1016/j.ijsu.2011.10.001 20332511 PMC2844943

[24] Ko J Kim M. Reliability and responsiveness of the gross motor function measure-88 in children with cerebral palsy. Phys Ther. (2013) 93(3):393–400. 10.2522/ptj.20110374 23139425

[25] Alonso-Coello P Schünemann HJ Moberg J Brignardello-Petersen R Akl EA Davoli M GRADE. Evidence to decision (EtD) frameworks: a systematic and transparent approach to making well informed healthcare choices. 1: introduction. Br Med J. (2016) 353:i2016. 10.1136/bmj.i2016 27353417

[26] Jackman M Sakzewski L Morgan C Boyd RN Brennan SE Langdon K Interventions to improve physical function for children and young people with cerebral palsy: international clinical practice guideline. Dev Med Child Neurol. (2022) 64(5):536–49. 10.1111/dmcn.15055 34549424

[27] Palisano R Rosenbaum P Walter S Russell D Wood E Galuppi B. Gross motor function classification system for cerebral palsy. Dev Med Child Neurol. (1997) 39(4):214–23. 10.1111/j.1469-8749.1997.tb07414.x 9183258

[28] Russell DJ Rosenbaum PL Cadman DT Gowland C Hardy S Jarvis S. The gross motor function measure: a means to evaluate the effects of physical therapy. Dev Med Child Neurol. (1989) 31(3):341–52. 10.1111/j.1469-8749.1989.tb04003.x 2753238

[29] Ferre-Fernández M Murcia-González MA Ríos-Díaz J. Intra- and interrater reliability of the Spanish version of the GMFM (GMFM-SP-88). Pediatr Phys Ther. (2022) 34(2):193–200. 10.1097/PEP.0000000000000874 35184079

[30] Norkin CC White DJ. Measurement of Joint Motion: A Guide to Goniometry. Philadelphia, PA: FA Davis (2016).

[31] Mutlu A Livanelioglu A Gunel MK. Reliability of ashworth and modified ashworth scales in children with spastic cerebral palsy. BMC Musculoskelet Disord. (2008) 9:44. 10.1186/1471-2474-9-44 18402701 PMC2330046

[32] World Health Organization. WHO Guidelines on Physical Activity and Sedentary Behaviour. Geneva: World Health Organization (2020). 33369898

[33] Alvandi F Amini M Ghafarzadeh Namazi N. Factors affecting the independence level of 4–6-year-old children with cerebral palsy in activities of daily living. Iran J Child Neurol. (2023) 17(4):93–104. 10.22037/ijcn.v17i2.37401 38074928 PMC10704291

[34] Steinbok P. Outcomes after selective dorsal rhizotomy for spastic cerebral palsy. Childs Nerv Syst. (2001) 17(1-2):1–18. 10.1007/PL00013722 11219613

[35] Delgado E Cumplido C Ramos J Garcés E Puyuelo G Plaza A ATLAS 2030 Pediatric gait exoskeleton: changes on range of motion, strength and spasticity in children with cerebral palsy. A case series study. Front Pediatr. (2021) 9:753226. https://doi: 10.3389/fped.2021.753226 34900862 PMC8652111

[36] Ammann-Reiffer C Bastiaenen CH Meyer-Heim AD van Hedel HJ. Effectiveness of robot-assisted gait training in children with cerebral palsy: a bicenter, pragmatic, randomized, cross-over trial (PeLoGAIT). BMC Paediatr. (2017) 17(1):64. 10.1186/s12887-017-0815-y PMC5333417 28253887

[37] Digiacomo F Tamburin S Tebaldi S Pezzani M Tagliafierro M Casale R Improvement of motor performance in children with cerebral palsy treated with exoskeleton robotic training: a retrospective explorative analysis. Restor Neurol Neurosci. (2019) 37(3):239–44. 10.3233/RNN-180897 31177250

[38] Peri E Turconi AC Biffi E Maghini C Panzeri D Morganti R Effects of dose and duration of robot-assisted gait training on walking ability of children affected by cerebral palsy. Technol Health Care. (2017) 25(4):671–81. 10.3233/THC-160668 28436398

